# Peripheral Odontogenic Keratocyst Presenting as a Gingival Nodular Mass in the Anterior Mandible: A Case Report

**DOI:** 10.7759/cureus.79382

**Published:** 2025-02-20

**Authors:** Itsuki Hayashi, Makoto Toida

**Affiliations:** 1 Oral and Maxillofacial Surgery, Sugita Genpaku Memorial Obama Municipal Hospital, Obama, JPN

**Keywords:** anterior mandible, gingival mass, odontogenic cyst, odontogenic keratocyst, peripheral

## Abstract

Odontogenic keratocyst (OKC) is an odontogenic cyst with a high recurrence rate due to its tumor-like characteristics, such as proliferative activity and daughter cyst formation. OKC is generally regarded as an intraosseous lesion; it may rarely occur as a peripheral lesion.

We present the case of a 73-year-old man who presented with a nodular mass on the gingiva of his mandibular anterior teeth and complained of discomfort. Intraoral examination revealed a dark red nodular mass 1.5 cm in size on the gingiva of the left mandibular central incisor. The lesion was completely excised under local anesthesia. The histopathological diagnosis was OKC. There was no intervening bone wall between the oral mucosa and the cyst wall; thus, the lesion was considered a peripheral OKC. This case report confirms that peripheral OKC is rare and requires careful attention in the differential diagnosis of gingival lesions.

## Introduction

Odontogenic keratocyst (OKC) is derived from the odontogenic epithelium and lined by complex keratinized stratified squamous epithelium [1]. Although OKC is generally regarded as an intraosseous lesion, it may rarely occur as a peripheral lesion and mimic the clinical appearance of an adult gingival cyst [2]. OKC originates from remnants of the dental lamina (or the rest of Serres) [2]. Peripheral OKC was slightly more frequent in females (66.6%), maxillary incisors, and premolars [3]. The histopathologic diagnosis of OKC shows keratinizing stratified squamous epithelium with walls within the cyst cavity and daughter cysts in the surrounding connective tissue [1]. The main treatment options for OKC are excision or enucleation, but no clear treatment strategy has been defined [2].

The incidence of peripheral OKC is unknown. It is a rare disease, with a few case series reported in recent years [3]. We present the case of a 73-year-old male patient with peripheral OKC in the gingiva of the anterior mandible.

## Case presentation

**Figure 1:**
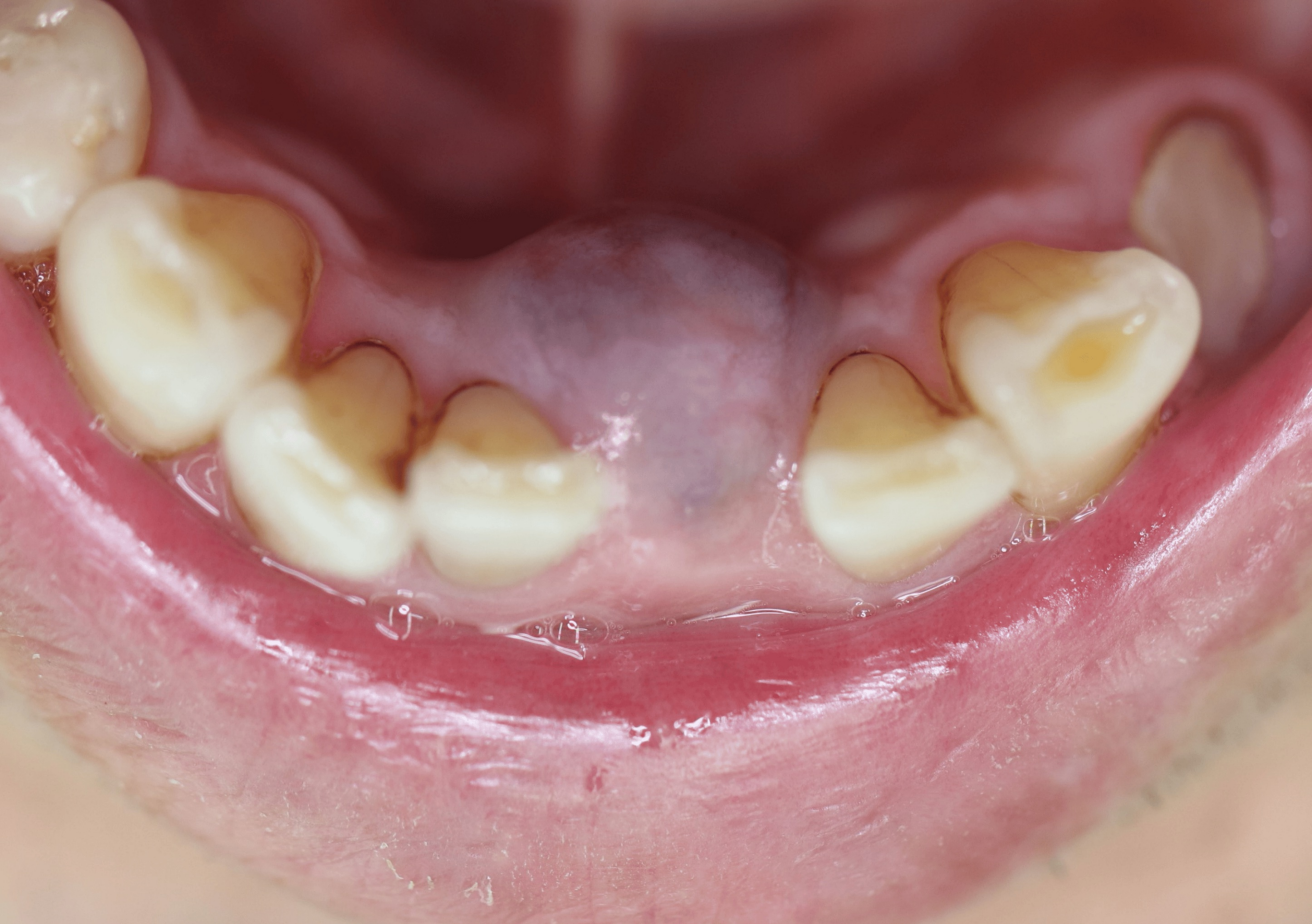
Image of a nodular mass in the gingiva of the anterior mandible at the time of initial examination.

A 73-year-old Japanese man presented with a nodular mass in the gingiva of the anterior mandible that had persisted for three months. The mass had gradually increased in size, and the patient was consequently referred to our department (Sugita Genpaku Memorial Obama Municipal Hospital, July 2019). Intraoral examination revealed a dark red nodular mass with a maximum dimension of 1.5 cm in the gingiva of the left mandibular central incisor region (Fig 1).

**Figure 2:**
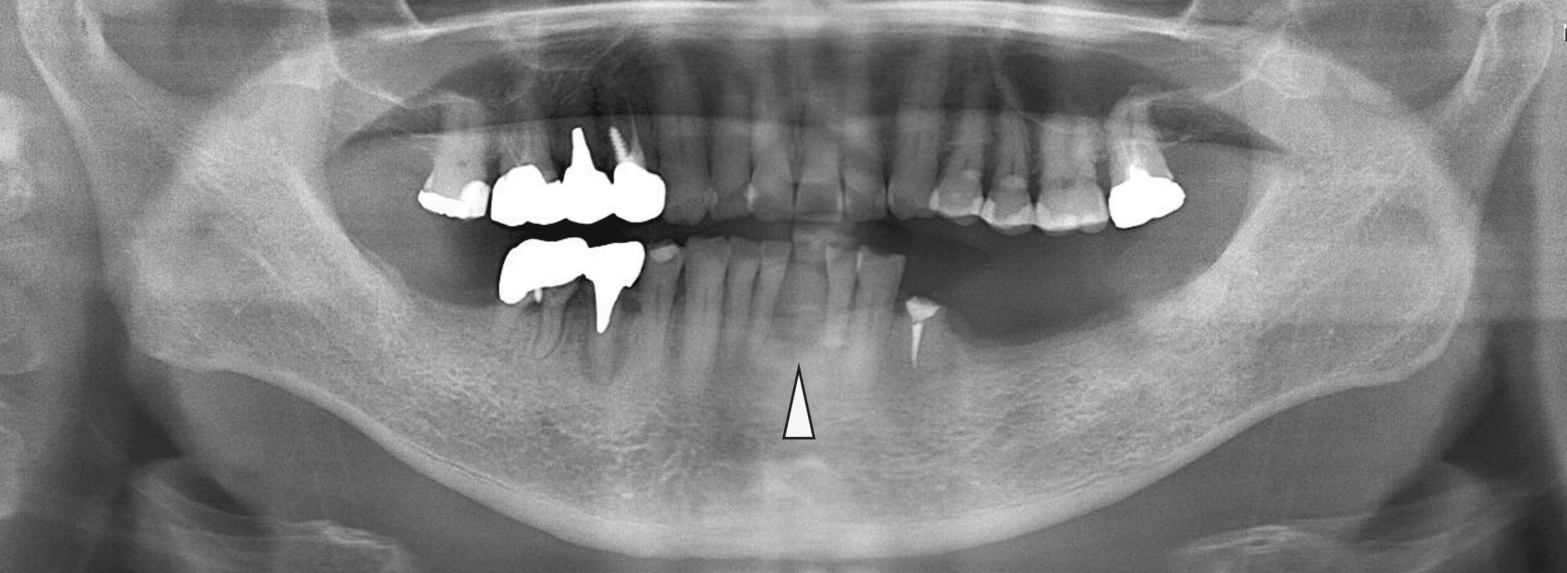
Preoperative panoramic radiograph A panoramic radiograph shows a well-defined mass with bone resorption localized in the missing left mandibular central incisor region (arrowhead).

**Figure 3:**
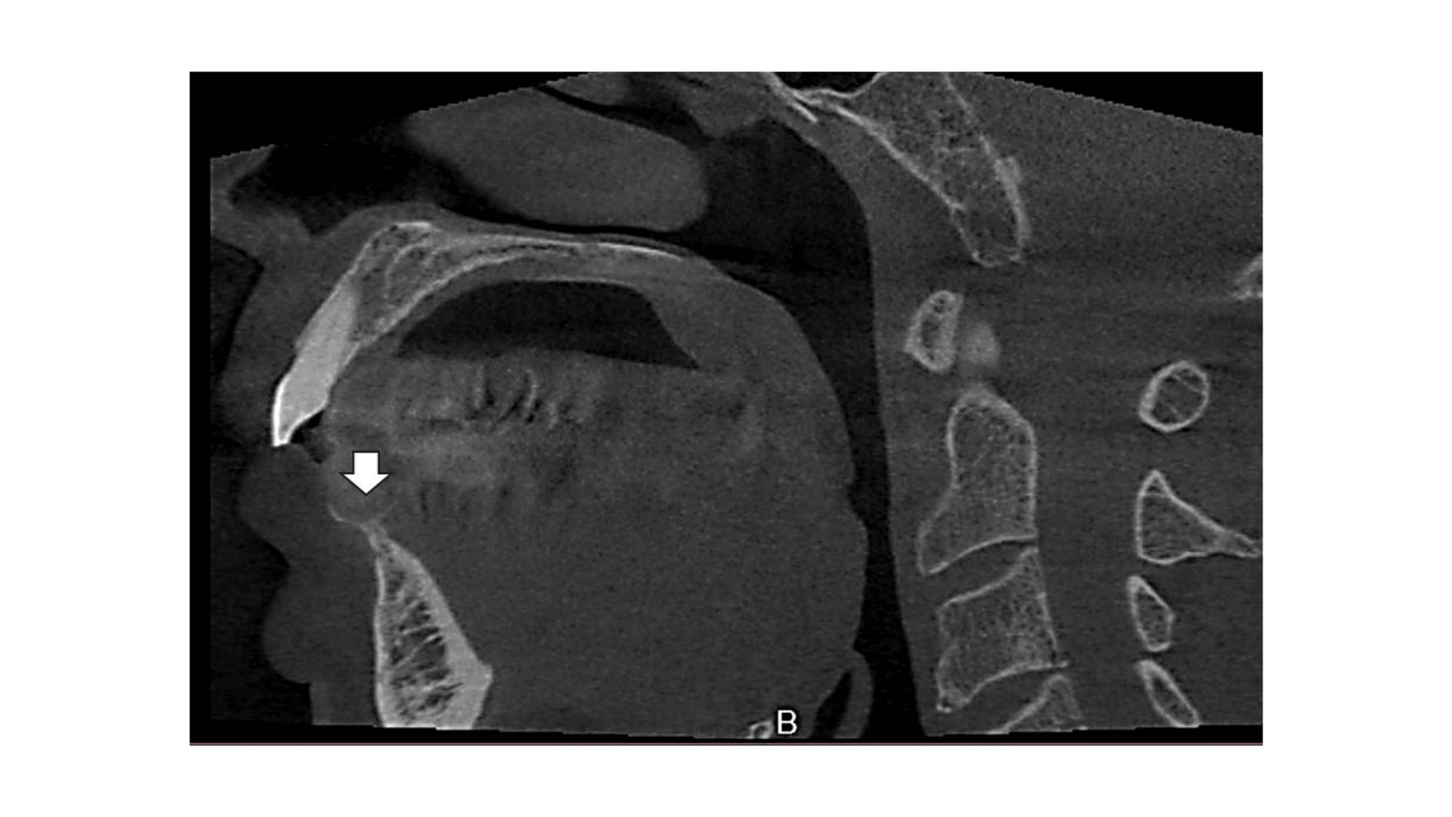
Preoperative computed tomography image A computed tomography image shows compressive bone resorption of the alveolar ridge owing to a lesion in the anterior mandibular region (arrow).

The patient stated that the tooth had been extracted many years prior to presentation. The gingival mass was soft, with a non-ulcerated smooth surface. Panoramic radiography and computed tomography (CT) revealed a well-demarcated elliptical radiolucent lesion confined to the missing left mandibular central incisor region, with compressive bone resorption due to the underlying bone lesion (Fig 2-3).

**Figure 4:**
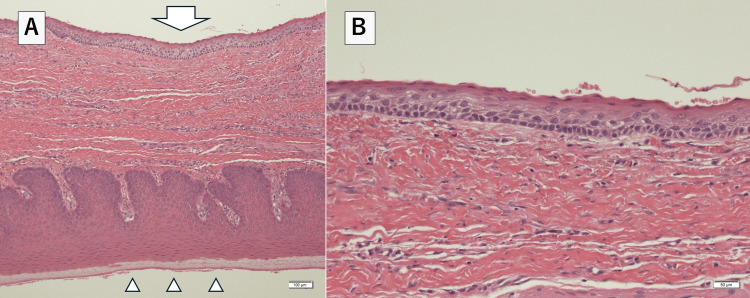
Histopathological examination image of the lesion (A): Microphotographs showing that the connective tissues of the cyst wall and the oral mucosa were continuous. Hematoxylin and eosin stain, scale bar=100μm; Arrow, cyst lining epithelium; arrowheads, oral mucosal epithelium. (B): The lesion presented para keratinized cyst epithelium and palisaded basal cells, which contain keratin in the cyst cavity. Hematoxylin and eosin stain, scale bar= 50μm

Clinical differential diagnoses included mesenchymal tumors, odontogenic tumors, and odontogenic cysts such as residual cysts. An incisional biopsy was performed under local anesthesia. After 0.5 ml of 2% adrenaline-added Xylocaine solution was administered to the margins of the lesion, the margins of the lesion were cut and biopsied with a scalpel. The histopathologic diagnosis was OKC. CT imaging findings showed compressive resorption of the surrounding bone, which suggested the possibility of local invasion. Therefore, we performed a marginal resection, including the tumor and surrounding bone. The resection area was set with a safety margin of about 3 mm from the tumor margins. The tumor and surrounding bone were removed in one lump using an ultrasonic cutting instrument. The wound was covered with an artificial membrane (polyglycolic acid sheet). Oral intake was resumed the day after surgery, and postoperative antimicrobials were administered for three days. The antibiotic was amoxicillin 750 mg for three days. Analgesics were prescribed, such as loxoprofen sodium hydrate 60 mg. Mouse rinses were prescribed with benzethonium chloride. A follow-up visit was conducted one week after the surgery. Microscopically, the lesion presented parakeratinized cyst epithelium and palisaded basal cells, which contained keratin in the cyst cavity (Fig 4).

**Figure 5:**
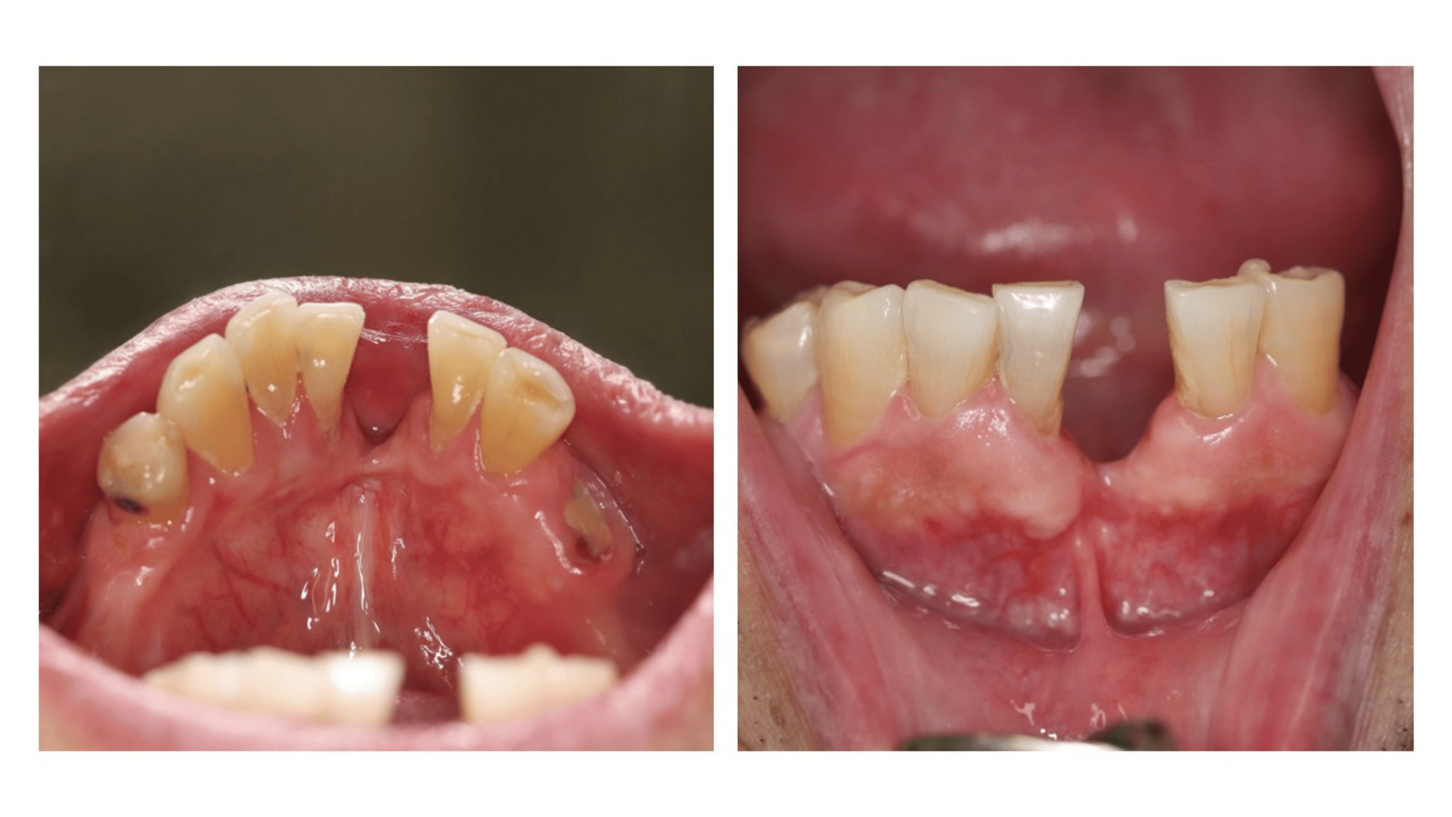
Intraoral view three years postoperatively, showing the wound completely covered with epithelium.

**Figure 6:**
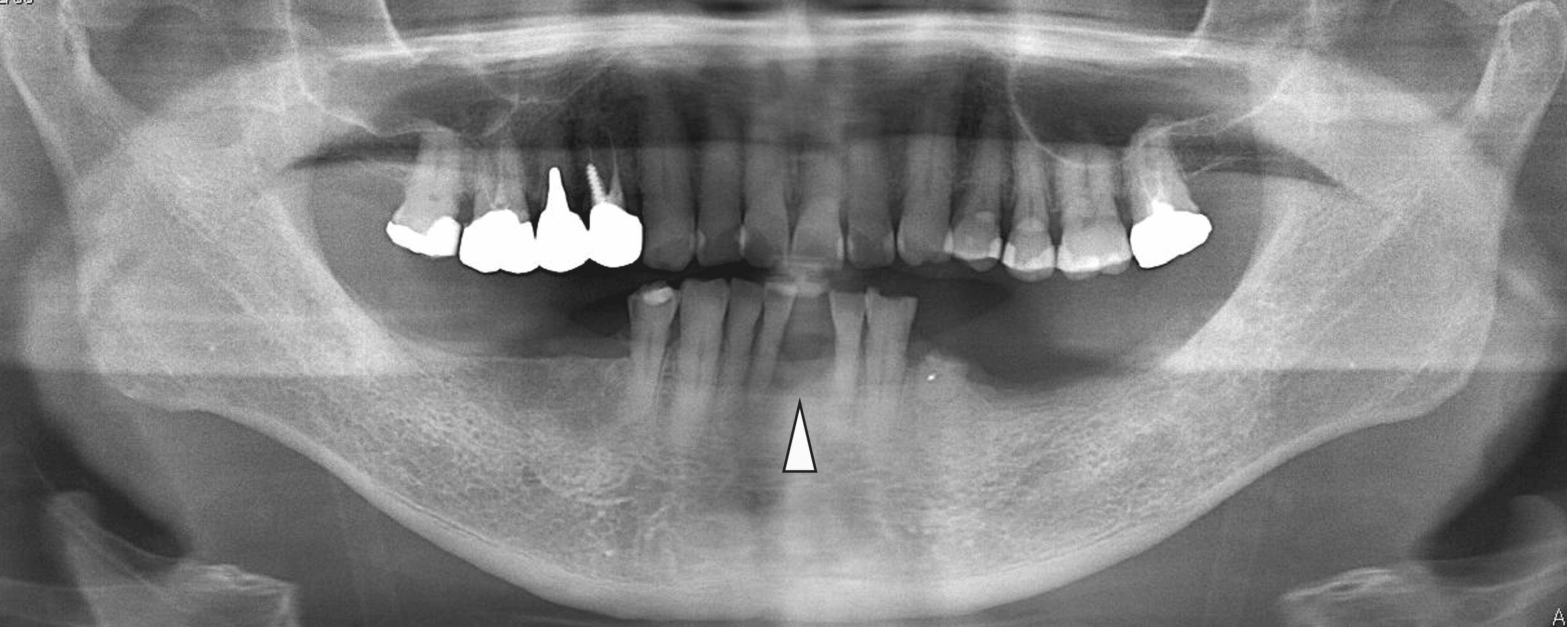
Postoperative panoramic radiograph No evidence of bone resorption is suspicious of recurrence (arrowhead).

The final histopathologic diagnosis was OKC. Furthermore, the lesion was considered peripheral OKC because there was no intervening bone wall between the oral mucosa and the cyst wall. Three years have passed since the surgery, and the patient is doing well with no evidence of recurrence (Fig 5-6).

## Discussion

Peripheral OKC was slightly more frequent in females (66.6%), maxillary incisors, and premolars (60%) [3]. The lesions are usually located on the vestibular gingiva and rarely on the palatal or lingual gingiva (5.5%) [3,4]. Tumor coloration is generally reported to be yellow, with pink and purple being less [5,6]. Furthermore, they are asymptomatic with no subjective symptoms [3].

The present lesion occurred on the alveolar apex of a mandibular anterior tooth and was dark red in color, suggesting that it was an extremely rare condition. The possible differential diagnosis of peripheral OKC is odontogenic cysts and tumors, as well as non-odontogenic tumors.

The characteristic imaging findings of peripheral OKC are unknown, but it has been reported that the bone surface in contact with the lesion is often resorbed [7]. Resorption of cortical bone by the lesion was observed in 45.8% of cases, and radiographic findings similar to lateral periodontal cysts have been reported in some cases [5].

Peripheral OKC should be included in the differential diagnosis of extraosseous nodular lesions because OKC can also occur in soft tissues [8]. The present lesion was considered a peripheral OKC because the bony surface tangential to the lesion was resorbed, and there was no intervening bone wall between the cyst wall and the oral mucosa.

The histopathology of peripheral OKC is similar to that of common endosteal OKC, with thin stratified squamous epithelium with a wavy parakeratinized surface, a nuclear palatal basal cell layer with cuboidal to columnar cells, keratin-like material in the cyst lumen, minimal inflammatory cell infiltration, and daughter cyst formation within connective tissue [9,10]. The present lesion also had the general findings of intraosseous type OKC. However, the cyst wall was completely within the connective tissue of the oral mucosa and was determined to be peripheral OKC. In addition to intraoral and imaging findings, histopathological findings based on tissue sampling are important in diagnosing this disease. The most common treatment for peripheral OKC is excision and curettage, with conservative surgery being the primary treatment of choice [9].

The recurrence rate of peripheral OKC is lower than that of intraosseous OKC, but local recurrence has been reported in 12.5-31.3% of cases [5,11]. However, no cases of malignant transformation have been reported [5]. Therefore, some reports recommend resectioning the tumor, including the periosteum [5]. Surgical removal with posterior curettage and a slight bone drilling of the area shows a low recurrence rate (17%) [9].

In the present case, since compressive resorption of alveolar bone was observed, a marginal resection was performed considering the possibility of intraosseous involvement of the lesion. There was no evidence of recurrence for three years postoperatively; long-term follow-up is planned. There have been few reports of peripheral OKC; this is the first report of a unique condition like this one. However, the validity of the treatment method needs to be established by accumulating more cases in the future.

## Conclusions

Peripheral OKC has a relatively high recurrence rate, as does the common intraosseous type of OKC. Existing reports show scattered cases of recurrence in patients who underwent excisional curettage. The present case demonstrated that marginal resection, including the surrounding bone, is extremely useful in controlling the lesion. Further case series are needed to determine the standard of care for peripheral OKC.
